# Platelet Receptor Glycoprotein VI-Dimer Is Overexpressed in Patients with Atrial Fibrillation at High Risk of Ischemic Stroke

**DOI:** 10.1055/s-0043-1776328

**Published:** 2023-11-13

**Authors:** Isuru Induruwa, Carly Kempster, Patrick Thomas, Harriet McKinney, Jean-Daniel Malcor, Arkadiusz Bonna, Joana Batista, Kenji Soejima, Willem Ouwehand, Richard W. Farndale, Kate Downes, Masaaki Moroi, Stephanie M. Jung, Elizabeth A. Warburton

**Affiliations:** 1 Department of Clinical Neurosciences, University of Cambridge, Cambridgeshire, United Kingdom of Great Britain and Northern Ireland; 2 Department of Haematology, University of Cambridge, Cambridgeshire, United Kingdom of Great Britain and Northern Ireland; 3 Department of Biochemistry, University of Cambridge, Cambridgeshire, United Kingdom of Great Britain and Northern Ireland; 4 Research and Development Coordination and Administration Department, KM Biologics Co., Ltd., Cambridgeshire, United Kingdom of Great Britain and Northern Ireland

**Keywords:** atrial fibrillation, platelets, thrombosis, ischemic stroke

## Abstract

**Introduction** Atrial fibrillation (AF) increases the risk of ischemic stroke (IS). We hypothesized that the functional form of platelet receptor glycoprotein (GP) VI, GPVI-dimer, which binds to collagen and fibrin causing platelet activation, is overexpressed in patients with AF who have not had a stroke.

**Methods** A total of 75 inpatients with AF were recruited. None were admitted with or had previously had thrombotic events, including IS or myocardial infarction. Platelet surface expression of total GPVI, GPVI-dimer, and the platelet activation marker P-selectin were quantitated by whole blood flow cytometry. Serum biomarkers were collected in AF patients. Results were compared against patients contemporaneously admitted to hospital with similar age and vascular risk-factor profiles without AF (noAF, *n* = 30).

**Results** Patients with AF have similar total GPVI surface expression ( *p* = 0.58) and P-selectin exposure ( *p* = 0.73) on their platelets compared with noAF patients but demonstrate significantly higher GPVI-dimer expression ( *p* = 0.02 *).* Patients with paroxysmal AF express similar GPVI-dimer levels compared with permanent AF and GPVI-dimer levels were not different between anticoagulated groups. Serum N-terminal pro b-type natriuretic peptide ( *p* < 0.0001 *)* and high sensitivity C-reactive protein ( *p* < 0.0001 *)* were significantly correlated with GPVI-dimer expression in AF platelets. AF was the only vascular risk factor that was independently associated with higher GPVI-dimer expression in the whole population ( *p* = 0.02 *)* .

**Conclusion** GPVI inhibition is being explored in clinical trials as a novel target for IS treatment. As GPVI-dimer is elevated in AF patients' platelets, the exploration of targeted GPVI-dimer inhibition for stroke prevention in patients at high risk of IS due to AF is supported.

## Graphical abstract

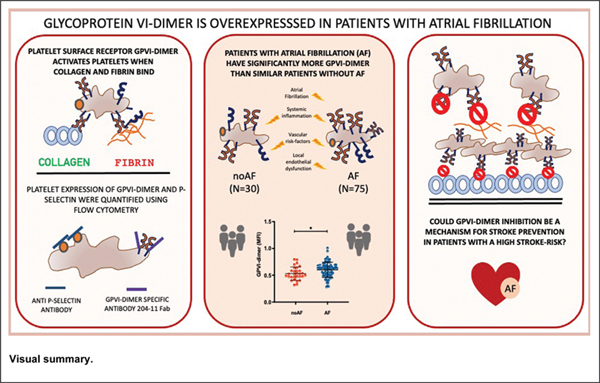

## Introduction

Atrial fibrillation (AF) is the most commonly encountered cardiac arrhythmia and can increase an individual's risk of ischemic stroke by up to 15% per year. 1 Even in the absence of vascular risk factors, the lifetime risk of developing AF is 20% 2 and as a result its incidence and prevalence are rising worldwide. 3 Ischemic strokes from AF (cardioembolic stroke [CES]) are severe and disabling 4 and guidelines recommend anticoagulation, rather than antiplatelets, to prevent CES. However, there is a risk of associated hemorrhage from these medicines, particularly in the older population. Therefore, there is a need for novel pharmacological targets with a favorable bleeding profile, aimed at preventing CES with AF.

Platelet receptor glycoprotein (GP) VI is one such target under investigation for future antithrombotic development in ischemic stroke, particularly as its inhibition is hypothesized to convey a low risk of hemorrhage. 5 GPVI is expressed on the platelet surface as a monomer or dimer; however, its functional form is the GPVI-dimer, accounting for approximately 29% of the total GPVI on circulating platelets. 6 GPVI-dimer has long been established as the platelet-collagen receptor, 7 whereby at sites of atherosclerotic plaque rupture, it binds to newly exposed subendothelial collagen and initiates intra-platelet signaling to cause activation, shape change, and recruitment of nonactivated platelets to the growing thrombus. 8 Recently, a novel interaction between GPVI and fibrin(ogen) has been recognized, where GPVI assists platelet activation through fibrinogen and fibrin binding, 9 10 and studies have demonstrated that this is a key interaction for thrombus stability and growth. 11 Furthermore, GPVI-dimers have been demonstrated to specifically bind fibrin fibers within in vitro formed clots 12 and in patients, CES thrombi retrieved at thrombectomy are platelet- and fibrin-rich, 13 suggesting that the platelet–fibrin interaction is an important contributor to the development of CES. This indicates a novel role for GPVI-dimer in CES, where AF causes low-velocity blood flow within the cardiac left atrial appendage, driving endothelial activation, tissue factor release, fibrin deposition, thrombus formation, and subsequent embolization to the brain.

We have recently reported that both total GPVI (monomeric and dimeric GPVI) and GPVI-dimer are overexpressed on the platelet surface in ischemic stroke patients at admission and at day-90 post-stroke. 14 Our primary objective with the *GRAFITE* study was to determine whether patients with AF who have not had a stroke also express more GPVI on their platelet surface and have more active circulating platelets, as measured by surface exposure of platelet P-selectin, compared with hospitalized patients with a similar age and vascular risk-factor profile who do not have AF (noAF). The secondary objectives were to determine correlations between GPVI-dimer expression and serum biomarkers in AF patients as well as risk factors within the whole population that may contribute to higher GPVI expression.

## Patients and Methods

The *GRAFITE* study was performed at a teaching hospital in Cambridge, United Kingdom between 2017 and 2020, where around 1,200 general medical patients per month are admitted via the emergency department (ED; AF prevalence 21.3% [2018–2019]). The AF cohort was individuals admitted with AF as a main diagnosis or a comorbidity, recruited while they were an inpatient, from medical inpatient wards. AF was diagnosed by admission 12-lead electrocardiogram (ECG) or on bedside cardiac monitoring. Paroxysmal AF (pAF) patients were eligible for participation in the study if the patient was in AF at the time of recruitment, checked by ECG or bedside cardiac monitoring. Patients taking anticoagulation were included in the study. The study protocol was approved by the Cambridge East Research Ethics Committee (16/EE/0436) and the inclusion and exclusion criteria can be found in the Supplementary Material.

## Comparator Population Recruitment

The comparator population (noAF) consisted of patients recruited contemporaneously, initially attending ED with stroke-like symptoms but then subsequently given a nonvascular diagnosis by the stroke team after appropriate investigations were completed ( Supplementary Table S1 ). Separately, *n* = 299 healthy donors without previous thrombotic disease (AF, ischemic heart disease (IHD), myocardial infarction, or stroke), known platelet disorders, or malignancy were invited and contemporaneously recruited from the National Institute for Health Research Cambridge BioResource (Cambridge East Ethics Committee (ref 10/H0304/65)). The GPVI-dimer and P-selectin comparisons between healthy donors and AF patients are presented in Supplementary Table S2 .

## Clinical Data Collection

For all participants, age, sex, history of relevant clinical risk factors (hypertension, congestive cardiac failure, diabetes mellitus, hypercholesterolemia, IHD, and history of previous thrombotic events including stroke) and medications at admission were collected. For noAF and AF cohorts, the national early warning score (NEWS) and CHA _2_ DS _2_ -VASc scores were calculated at the time of recruitment and electronic medical records were checked at 6 months to clarify further vascular events or death.

## Flow Cytometry Quantification of GPVI-Dimer Expression and Resting P-selectin Exposure

All participants were consented to give 30 to 50 mL of whole blood for analysis. Blood collection methods are detailed in the Supplementary Material and quantification of GPVI expression and P-selectin exposure using whole blood was performed as previously. 14 Total GPVI and GPVI-dimer present on the platelet surface were calculated as mean fluorescence intensity (MFI). Anti-total-GPVI HY101 (12.5 µg/mL; IBGRL Bristol, United Kingdom) was used for total GPVI quantification and anti-GPVI-dimer 204–11 Fab (5 μg/mL), a nonactivating, noninhibitory Fab developed by M.M. and S.M.J. (in collaboration with K.S., Kumamoto, Japan), was used for GPVI-dimer quantification. 15 These antibodies bind to distinct areas of platelet surface GPVI, allowing separate quantification of total (monomeric and dimeric [HY101]) as well as dimeric GPVI (204–11 Fab).

Platelet activation was measured by detecting platelet surface expression of the activation marker P-selectin (also known as CD62p) 16 using PE-conjugated anti-CD62P at a dilution of 1:50, calculated as % positive platelets (%PP). Platelets were identified by light scatter and results were recorded as the percentage of platelets positive (%PP) for P-selectin, which was calculated as the percentage of platelets expressing P-selectin with MFI greater than 98% of the isotype control. Flow cytometry analyses for all samples were performed in the same laboratory by the same personnel for all patients and healthy donors in the study.

## Serum Biomarker Analysis

Separate whole blood samples were collected at the same time in AF patients for routine hematology and biochemical tests: full blood count and international normalized ratio as well as serum biomarker analysis: D-dimer, fibrinogen, N-terminal pro b-type natriuretic peptide (BNP), and high-sensitivity C-reactive protein (hs-CRP). These tests were performed by the Cambridge University Hospital Biochemistry and Pathology departments and normal ranges are presented in the Supplementary Material.

## Statistical Analysis

A *p-* value of ≤ 0.05 was taken as statistically significant. A power calculation determined that <20 participants were required in each group (type I error of 0.05 and power of 0.80). Shapiro–Wilk testing was performed for determining normal distributions prior to statistical analysis. Comparisons were performed using *t* -test for parametric data, the Mann–Whitney U test for nonparametric data, and Chi-squared for categorical data. Correlation was calculated using Spearman's rank correlation co-efficient for nonparametric data. Simple linear regression analysis was used to establish associations between GPVI-dimer expression and other single predictor variables. Unstandardized coefficient (B) and significance ( *p* ) are reported for each of the significantly associated variables. Data were analyzed using Prism v9.1.1 (GraphPad, San Diego, California, United States) and SPSS v.29 (IBM Corp., Armonk, New York, United States).

## Results

A total of 79 patients with AF were recruited, but 4 had to be excluded (1 on heparin infusion at recruitment, 3 due to incomplete GPVI-dimer or P-selectin data), leaving 75 patients for final analysis. A total of 30 patients who presented with stroke-like symptoms but were subsequently diagnosed with a nonvascular cause who did not have a history of AF make up the noAF cohort ( Table 1 ). The mean time ± standard deviation (SD) between venipuncture and start of processing was 20 ± 5 minutes in the AF group, and 32 ± 7 minutes in the noAF group and all patients were recruited in the first 24 hours of admission to hospital.

**Table 1:** Baseline characteristics of the no AF and AF populations

	No AF	AF	*p*
*n*	30	75	–
Median age (Q _1_ –Q _3_ )	76 (60–85)	74 (66–79)	0.55
Female (%)	60.0	38.7	0.08
Known AF (%)	Excluded	84.0	–
Mean hemoglobin (g\L) ± SD	137.3 ± 15.6	134.0 ± 20.2	0.42
Mean platelet count (10 × 9/L) ± SD	247.4 ± 60.1	247.0 ± 80.1	0.98
Mean platelet volume (fL) ± SD	10.7 ± 0.9	10.6 ± 1.0	0.76
Median admission NEWS score (Q _1_ –Q _3_ )	1 (0–2)	1 (1–2)	0.15
Deaths within 6 months (%)	0 (0)	0 (0)	–
Vascular events within 6 months (%)	0 (0)	0 (0)	–
Risk factors for thrombotic disease, *n* (%)			
Congestive cardiac failure	6 (20.0)	22 (29.3)	0.46
Hypertension	16 (53.3)	51 (68.0)	0.24
Diabetes	7 (23.3)	19 (25.3)	0.83
Ischemic heart disease	6 (20.0)	14 (18.6)	0.88
Hypercholesterolemia	8 (26.7)	31 (41.3)	0.24
Stroke in past 10 years	0 (0)	0 (0)	–
Median CHA _2_ DS _2_ -VASc score	3 (1–5)	3 (2–4)	0.92
Admission medication, n (%)			
ACE inhibitor or ARB	8 (26.7)	28 (37.3)	0.42
Aspirin	5 (16.7)	14 (18.6)	0.81
Clopidogrel	6 (20.0)	3 (4.0)	**0.02**
Apixaban	1 (3.3)	9 (12.0)	0.32
Dabigatran	0 (0)	1 (1.3)	0.50
Rivaroxaban	0 (0)	25 (33.3)	**0.0008**
Warfarin	0 (0)	12 (16.0)	0.05
Statin	10 (30.0)	34 (45.3)	0.36
Serum biomarkers, median (Q _1_ –Q _3_ )			
Median d-dimer (ng/mL)	–	158 (80–387)	–
Median fibrinogen (g/L)	–	3.71 (2.89–4.72)	–
Median BNP (pg/mL)	–	2254 (623–4467)	–
Median hs-CRP (mg/L)	–	24.8 (16.3–30.6)	–

Abbreviations: ACE, angiotensin-converting enzyme; ARB, angiotensin receptor blocker; BNP, N-terminal pro b-type natriuretic peptide; hs-CRP, high-sensitivity C-reactive protein.

Note: Median values are presented with interquartile range (Q _1_ –Q _3_ ) and mean values with standard deviation (SD). *p* -Values were calculated using a paired *t* -test for parametric and Mann–Whitney U test for nonparametric continuous data and Chi-squared test for categorical data. Statistically significant *p* -values are indicated in bold.

Patients in the noAF and AF cohorts were evenly matched by age, (median years (Q _1_ –Q _3_ ; noAF: 76 (60–85), AF: 74 (66–79), *p* = 0.55) and risk factor profile ( Table 1 ). There was no difference between the clinical acuity scores at venipuncture between the two groups and none of the patients died within 6 months of data collection.

## AF Patients Express Significantly more GPVI-Dimer Compared with Patients without AF

**Fig. 1:**
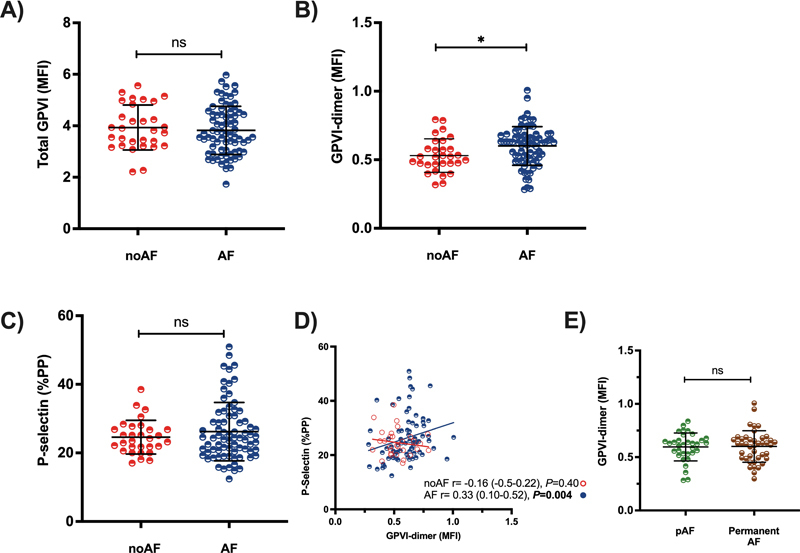
( **A** ) **Total GPVI,** ( **B** ) **GPVI-dimer expression, and** ( **C** ) **resting P-selectin exposure between noAF (** ***n*** **= 30) and AF cohorts (** ***n*** **= 75).** The error bars represent the mean MFI ± SD or the median (Q1–Q3) %PP. ( **D** ) Correlation between GPVI-dimer and P-selectin in noAF and AF patients. These data were calculated using Spearman's rank correlation coefficient. Blue regression line = AF, red regression line= noAF. ( **E** ) GPVI-dimer expression between patients with pAF and permanent AF. The error bars represent the mean MFI of each of the cohorts ± SD. AF, atrial fibrillation; GPVI, glycoprotein VI; MFI, mean fluorescence intensity; ns, not significant; pAF, paroxysmal atrial fibrillation; %PP, percentage of platelets positive; SD, standard deviation.

AF patients' platelets expressed significantly higher GPVI-dimer (MFI ± SD; noAF: 0.53 ± 0.12, AF: 0.60 ± 0.14, *p* = 0.02; Fig. 1B ) compared with noAF. However, total GPVI (MFI ± SD; noAF: 3.93 ± 0.87, AF: 3.82 ± 0.94, *p* = 0.58; Fig. 1A ) and resting P-selectin exposure patients were similar compared with noAF (median %PP (Q _1_ –Q _3_ ); noAF: 23.9 (21.5–27.1), AF: 24.4 (20.4–30.8), *p* = 0.73) ( Fig. 1C ). Furthermore, GPVI-dimer expression ( *p* = 0.04) and P-selectin exposure ( *p* < 0.0001) were significantly higher in the AF patients compared with healthy donors ( Supplementary Table S2 ). GPVI-dimer expression in patients with AF correlated to resting P-selectin exposure ( *r* = 0.33, *p* = 0.004; Fig. 1D ), whereas they did not in noAF patients ( *r* = − 0.16, *p* = 0.40; Fig. 1D ), indicating a unique relationship between GPVI-dimer formation and P-selectin exposure in AF patients' platelets.

## Paroxysmal AF Patients Express Similar GPVI-Dimer Levels Compared with Permanent AF

In total, 33/75 patients had pAF. pAF patients were significantly younger ( *p* = 0.0004) but had similar CHA _2_ DS _2_ -VASc scores, suggesting a comparable risk of future ischemic stroke ( *p* = 0.26; Table 2 ), compared with permanent AF patients. They expressed similar GPVI-dimer levels (MFI ± SD; pAF = 0.60 ± 0.13; permanent AF = 0.60 ± 0.16, *p* = 0.80; Fig. 1E ) but significantly less resting P-selectin exposure compared with those in permanent AF (median (Q _1_ –Q _3_ ) (%PP); pAF: 22.5 (18.9–26.7), permanent AF: 25.7 (21.4–35.0), *p* = 0.009).

**Table 2:** Characteristics of the paroxysmal AF and permanent AF patients

	Paroxysmal AF	Permanent AF	*p*
*n*	33	42	
Mean age (y) ± SD	67.5 ± 10.1	75.3 ± 8.2	**0.0004**
Female (%)	8 (25.8)	21 (50.0)	**0.03**
On anticoagulation (%)	19 (57.6)	31 (73.8)	0.14
Risk factors for thrombotic disease, *n* (%)			
Congestive cardiac failure	7 (21.2)	15 (35.7)	0.21
Hypertension	22 (66.7)	29 (69.0)	1.0
Diabetes	12 (36.4)	7 (16.7)	0.06
Ischemic heart disease	4 (12.2)	10 (23.8)	0.24
Cholesterol	17 (51.5)	14 (33.3)	0.16
Median CHA _2_ DS _2_ -VASc score	3 (2–4)	3 (2–5)	0.26

## GPVI-Dimer Expression Is Correlated with BNP and hs-CRP in AF Patients

**Fig. 2:**
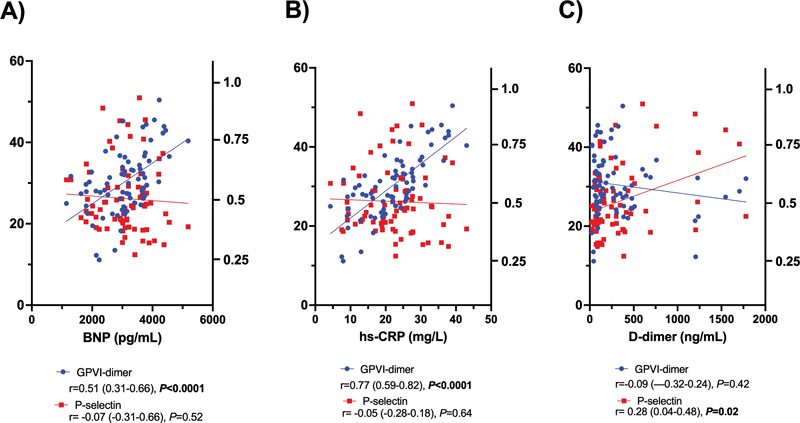
**Correlation between GPVI-dimer expression or P-selectin exposure and** ( **A** ) **BNP,** ( **B** ) **hs-CRP, and** ( **C** ) **D-dimer in patients with AF.** Correlations were calculated using Spearman's rank correlation coefficient. Regression line is presented in blue (GPVI-dimer) or red (P-selectin). BNP, N-terminal pro b-type natriuretic peptide; hs-CRP, high-sensitivity C-reactive protein.

GPVI-dimer expression in AF patients demonstrated a strong positive correlation with serum BNP ( *r* = 0.51, *p* < 0.0001; Fig. 2A ) and hs-CRP ( *r* = 0.72, *p* < 0.0001; Fig. 2B ). Neither serum D-dimer ( *p* = 0.42) or fibrinogen ( *p* = 0.88) was correlated with GPVI-dimer. P-selectin exposure was only correlated with D-dimer ( *r* = 0.28, *p* = 0.02; Fig. 2C ) and none of the serum biomarkers were correlated with total GPVI expression.

## AF is a Predictor of GPVI-dimer Expression

Associations between single-predictor variables with total GPVI and GPVI-dimer were performed using simple linear regression in the whole population of AF and noAF patients. AF was independently associated with higher GPVI-dimer expression ( Table 3 ; B = 0.07, *p* = 0.02).

**Table 3:** Single predictor variables associated with total GPVI and GPVI-dimer expression in the whole population of AF and noAF patients ( *n* = 105)

	Total GPVI		GPVI-dimer	
	Coefficient B	Significance ( *p* )	Coefficient B	Significance ( *p* )
Age	−0.03	0.10	−0.01	0.30
Atrial fibrillation	−0.09	0.62	**0.07**	**0.02**
Congestive cardiac failure	−0.26	0.28	0.03	0.45
Hypertension	0.26	0.18	−0.004	0.89
Diabetes	0.29	0.16	0.03	0.92
Ischemic heart disease	−0.10	0.65	0.04	0.25
High cholesterol	−0.007	0.97	0.006	0.83

Abbreviation: GPVI, glycoprotein VI.

## Discussion

Circulating platelets are subject to a constant stream of activating signals through their platelet surface receptors, a process called “platelet priming.” Therefore, the understanding of such receptors, their role in thrombosis, and their downregulation as a mechanism to prevent or treat thrombotic disease is crucial. Elevated expression and activity of key platelet receptors such as GPVI have been demonstrated in acute coronary syndromes 17 and ischemic stroke, 14 18 with studies reporting higher GPVI expression with poorer clinical outcomes, 19 explaining the abundance of work being performed investigating its inhibition as a potential mechanism to reduce thrombosis.

This is the only study to have quantified and compared GPVI expression and P-selectin exposure in patients at high risk of ischemic stroke due to AF with a comparator population without AF, similar in age, vascular risk factors, and CHA _2_ DS _2_ -VASc scores. Both AF and noAF patients have similar total GPVI expression and circulating P-selectin exposure, which has been reported in other studies. 20 However, platelets from patients with AF demonstrate significantly higher surface expression of GPVI-dimer, the functional form of GPVI, compared with both noAF patients and healthy donors. The similarity in P-selectin levels between AF and noAF patients suggests that although both populations of patients have activated circulating platelets due to the presence of vascular risk factors, as they are both higher than P-selectin exposure of healthy donors, AF patients' platelets may be subject to other factors that separately cause GPVI dimerization, such as inflammation. 21

AF and inflammation are closely linked, where inflammation can lead to the pathogenesis of AF, and AF subsequently propagates both local and systemic inflammatory responses. 22 GPVI is also linked to inflammation, whereby sustained GPVI responses are able to form pro-inflammatory microparticles and initiate pro-coagulant responses within the circulation. 23 GPVI blockade has also shown to downregulate inflammatory cell recruitment in animal models of stroke, 24 hence its recognition as a mediator of thromboinflammation. 25 Therefore, the higher GPVI-dimer expression in AF patients, compared with noAF, likely represents an AF-specific effect on platelets. This is further corroborated by the fact that total GPVI and GPVI-dimer were correlated in noAF platelets but not in AF platelets ( Supplementary Fig. S2 ), which may partly represent dimerization of platelet surface monomers with platelet activation due to AF.

Our data show that AF was the only risk factor independently associated with higher GPVI-dimer expression in the whole patient population and furthermore, BNP and hs-CRP, serum biomarkers linked with AF and inflammation, were significantly correlated with GPVI-dimer expression and not P-selectin exposure in AF patients. The similarity in GPVI-dimer expression between permanent AF and pAF further indicates that it is the presence, rather than the burden of AF, that contributes to GPVI-dimer expression. The fact that only AF patients' platelets demonstrated a strong correlation between GPVI-dimer expression and P-selectin exposure suggests a close link between AF, GPVI-dimerization, platelet activity, and the potential for thrombosis.

There was no difference between the markers of acute illness measured between the cohorts (NEWS score), and only 12/75 AF patients were admitted with an infection or sepsis, suggesting that higher dimer expression is constitutive to AF patients' platelets, rather than an acute reaction to an intercurrent illness.

The increased platelet surface expression of GPVI-dimer in patients with AF could be crucial in different ways, particularly in the context of ischemic stroke. Higher GPVI-dimer levels in AF patients' platelets would contribute to higher affinity binding to fibrin(ogen) leading to thrombus growth and increased thrombus stability. This would be deleterious in conditions that favor thrombogenesis, such as within the cardiac left atrial appendage, the nidus for thrombus formation in AF that leads to CES. 26 Drugs in development aimed at inhibiting GPVI-dimer in stroke are demonstrating promising results. 27 It could be hypothesized that molecules such as glenzocimab, a humanized fragment of antibody (Fab) against platelet GPVI, which is able to cause thrombus disaggregation by interrupting GPVI–fibrin interactions, 11 may have a role to play in downregulating some of the GPVI–fibrin interactions in AF patients. Other studies employing Fab blockade of GPVI have demonstrated similar results, highlighting the importance of GPVI–fibrin interactions in maintaining thrombus integrity during the early stages of thrombosis. 28

Interestingly, we found no difference in GPVI expression between anticoagulated and nonanticoagulated AF patients ( Supplementary Fig. S1 ), a finding consistent with previous published research. 14 29 This further highlights that patients with AF who are at risk of CES and have higher levels of circulating GPVI-dimer on their platelets could be a population of patients where GPVI inhibition may play a decisive role.

There are some limitations to this study: we did not collect serum biomarkers in noAF patients, which would have allowed us direct comparisons between GPVI-dimer expression and biomarkers between the two cohorts, further reinforcing the link to inflammation. The numbers in the noAF group are lower than that of the AF cohort, but they serve as an excellent comparator group due to their similarity in age, risk factors, and CHA _2_ DS _2_ -VASc scores. As expected, significantly higher GPVI-dimer expression, as well as P-selectin exposure, is noted when comparing the healthy donor population with AF patients.

## Conclusion

Ischemic stroke patients, regardless of stroke etiology, 14 and also patients with AF, without thrombotic disease, have demonstrated significantly higher platelet surface GPVI-dimer expression. GPVI-dimer has a key role in thrombus formation, growth, and stability using collagen and fibrin as ligands, as well as through inflammation-mediated responses. The inhibition of GPVI-dimer is being investigated as a pharmacological target for the treatment of ischemic stroke. Whether GPVI-dimer downregulation also has a role in reducing stroke risk in patients with AF, a population at high risk of ischemic stroke, needs investigation in future studies.

## Notes

**What Is Known about This Topic?**

- The platelet surface receptor glycoprotein (GP) VI-dimer has been long established as the collagen receptor on platelets, activating platelets at sites of plaque rupture.
- Activation of GPVI by fibrin contributes to thrombus growth and stabilization.
- Patients with ischemic stroke express more GPVI-dimer on their platelet surface than healthy controls.

**What Does This Paper Add?**

- Patients with a cardiac arrythmia called atrial fibrillation (AF), who are at high risk of ischemic stroke, express significantly more platelet GPVI-dimer compared with similar patients without AF and healthy donors.
- GPVI-dimer levels are correlated with serum biomarkers of inflammation in AF patients.
- GPVI-dimer inhibition should be explored as a novel target for potential stroke *prevention* in AF, as well as in acute stroke treatment.
