# Supplementary material for: Platelet Receptor Glycoprotein VI-Dimer Is Overexpressed in Patients with Atrial Fibrillation at High Risk of Ischemic Stroke
Source: TH Open. 2023 Nov 13;7(4):e294–302. doi: 10.1055/s-0043-1776328 (PMC10643047; doi:10.1055/s-0043-1776328)
Supplement: Supplementary file 1 — Supplementary Material [file 10-1055-s-0043-1776328-s23060025.pdf]

## Supplementary Material

### Exclusion Criteria

The exclusion criteria for healthy donor, atrial fibrillation (AF), and noAF groups were individuals <18 years of age, pregnancy, known past or active malignancy, platelet disorder or abnormal platelet count ($<150 \times 10^9$/L), hemoglobin (Hb: <90 g\L), human immunodeficiency virus, or hepatitis. Patients admitted with or had had a confirmed transient ischemic attack, myocardial infarction, or stroke in the past 10 years were also excluded from the study. Specifically for the AF cohort, no one without a confirmed diagnosis of AF or paroxysmal AF (pAF) was approached or recruited.

### Blood Collection

Healthy donor, noAF, and AF patient blood samples were drawn from the antecubital fossa vein using a 21-gauge butterfly needle. Blood was taken by stroke research nurses or by Dr. Induruwa for all patients and used a set protocol for collection as detailed below.

The first tube drawn was a K2 EDTA Vacutainer (BD, Franklin Lakes, New Jersey, United States), which was solely used for full blood count quantification. Next, blood was drawn into a 0.109 mol/L sodium citrate Vacuette (Greiner, Kremsmü nster, Austria). To avoid artifactual platelet activation, after removing the tourniquet, the first 5 mL was discarded, and blood was then taken to the laboratory on site for immediate analysis for healthy donor, AF, and noAF samples. A Sysmex XN-1000 (Sysmex, Kobe, Japan) was used to carry out a full blood count of all samples at the same time as glycoprotein VI (GPVI) and platelet function measurements. For AF patients, one EDTA, one citrate, and two serum Vacutainers were taken to the Cambridge University Hospital Biochemistry and Pathology departments for immediate analysis.

### Normal Ranges for Haematology and Biochemistry Tests

Platelet count: 150–450 10 × 9/L.
D-dimer: 0–230 ng/mL.
Fibrinogen: 1.46–3.33 g/L.
BNP < 100 pg/mL.
hs-CRP < 10 mg/L.

**Supplementary Table S1** Discharge diagnoses in noAF and AF patients

| | No AF | AF |
|---|---|---|
| AF-related symptoms | 0 | 39 |
| Infection/sepsis | 3 | 12 |
| Congestive cardiac failure | 0 | 8 |
| Migraine | 8 | 1 |
| Noncardiac chest pain | 1 | 7 |
| Syncope/fall | 5 | 3 |
| Seizures | 4 | 0 |
| Electrolyte disturbances | 0 | 3 |
| Unclear, nonvascular, no stroke on MRI | 3 | 0 |
| Viral illness | 2 | 1 |
| Hypoglycemia | 1 | 0 |
| Musculoskeletal | 1 | 1 |
| Vestibular symptoms | 2 | 0 |

Abbreviations: AF, atrial fibrillation; MRI, magnetic resonance imaging.

 © 2023. The Author(s).

**Supplementary Table S2** Baseline characteristics of the healthy donor and AF populations

| | Healthy Donor | AF | *p* |
|---|---|---|---|
| *n* | 299 | 75 | – |
| GPVI-dimer expression and P-selectin exposure | | | |
| Total GPVI, MFI ± SD | 3.97 ± 0.70 | 3.82 ± 0.94 | 0.09 |
| GPVI-dimer, MFI ± SD | 0.55 ± 0.14 | 0.60 ± 0.14 | **0.04** |
| Resting P-selectin, %PP ($Q_1$–$Q_3$) | 19.1 (16.2–21.7) | 24.4 (20.4–30.8) | $<$**0.0001** |
| Demographic details | | | |
| Median age ($Q_1$–$Q_3$) | 60 (47–68) | 74 (66–79) | $<$**0.0001** |
| Female (%) | 65.8 | 38.7 | $<$**0.0001** |
| Known AF (%) | Excluded | 84.0 | – |
| Mean hemoglobin (g\L) ± SD | 136.5 ± 11.4 | 134.0 ± 20.2 | 0.15 |
| Mean Plt count (10 × 9/L) ± SD | 259.6 ± 57.3 | 247.0 ± 80.1 | 0.11 |
| Mean Plt volume (fL) ± SD | 10.4 ± 0.9 | 10.6 ± 1.0 | **0.04** |
| Risk factors for thrombotic disease, *n* (%) | | | |
| Congestive cardiac failure | 0 (0) | 22 (29.3) | $<$**0.0001** |
| Hypertension | 26 (8.7) | 51 (68.0) | $<$**0.0001** |
| Diabetes | 1 (0.33) | 19 (25.3) | $<$**0.0001** |
| Ischemic heart disease | 0 (0) | 14 (18.6) | $<$**0.0001** |
| Hypercholesterolemia | 23 (7.7) | 31 (41.3) | $<$**0.0001** |
| Stroke in past 10 years | 0 (0) | 0 (0) | – |
| Median $CHA_2DS_2$-VASc score | 1 (1–2) | 3 (2–4) | $<$**0.0001** |
| Admission medication, *n* (%) | | | |
| ACE inhibitor or ARB | 20 (6.7) | 28 (37.3) | $<$**0.0001** |
| Aspirin | 0 (0) | 14 (18.6) | $<$**0.0001** |
| Clopidogrel | 0 (0) | 3 (4.0) | **0.02** |
| Apixaban | 0 (0) | 9 (12.0) | $<$**0.0001** |
| Dabigatran | 0 (0) | 1 (1.3) | 0.20 |
| Rivaroxaban | 0 (0) | 25 (33.3) | $<$**0.0001** |
| Warfarin | 0 (0) | 12 (16.0) | $<$**0.0001** |
| Statin | 22 (7.4) | 34 (45.3) | $<$**0.0001** |

Abbreviations: ACE, angiotensin-converting enzyme; ARB, angiotensin receptor blocker; AF, atrial fibrillation; BNP, N-terminal pro b-type natriuretic peptide; hs-CRP, high-sensitivity C-reactive protein; Plt, platelet.
Note: Median values are presented with interquartile range ($Q_1$–$Q_3$) and mean values with standard deviation (SD). *p*-Values were calculated using an unpaired *t*-test for parametric and Mann–Whitney U-test for nonparametric continuous data and Chi-squared test for categorical data. Serum biomarkers are presented with normal range and units. Statistically significant *p*-values are indicated in bold.

 © 2023. The Author(s).

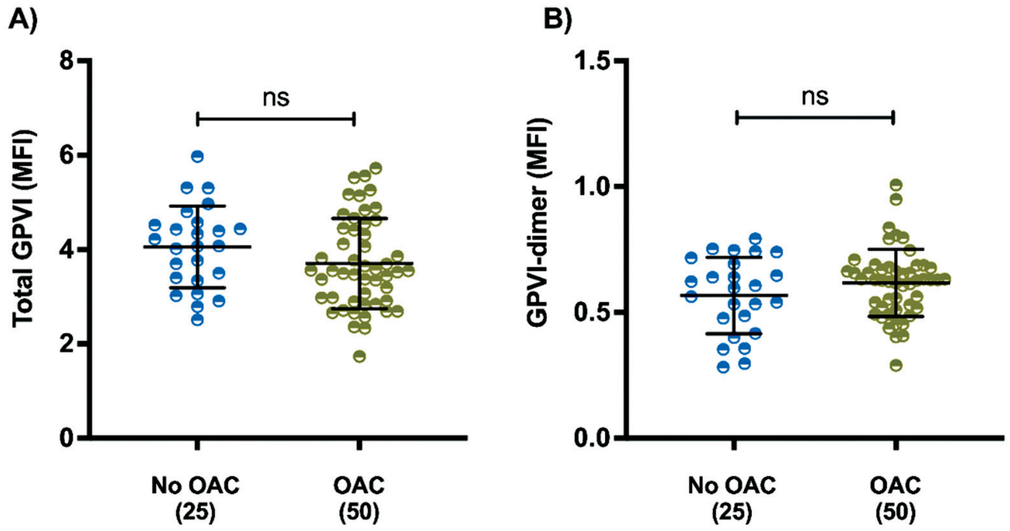


**Supplementary Fig. S1. (A) Total GPVI and (B) GPVI-dimer expression between not anticoagulated (No OAC, *n* = 25) and anticoagulated (OAC, *n* = 50) AF patients.** The error bars represent the mean MFI ± SD. GPVI, glycoprotein VI; MFI, mean fluorescence intensity; ns, not significant; OAC, anticoagulation.

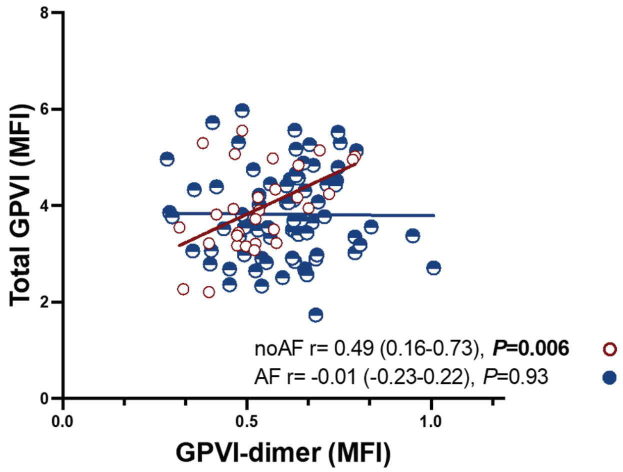


**Supplementary Fig. S2. Correlation between Total GPVI and GPVI-dimer expression in patients with AF (blue) or noAF (red).** Correlations were calculated using Spearman's rank correlation coefficient. GPVI, glycoprotein VI; MFI, mean fluorescence intensity.

 © 2023. The Author(s).
